# Replication-Deficient Particles: New Insights into the Next Generation of Bluetongue Virus Vaccines

**DOI:** 10.1128/JVI.01892-16

**Published:** 2016-12-16

**Authors:** Cristina C. Celma, Meredith Stewart, Kerstin Wernike, Michael Eschbaumer, Lorenzo Gonzalez-Molleda, Emmanuel Breard, Claudia Schulz, Bernd Hoffmann, Andy Haegeman, Kris De Clercq, Stephan Zientara, Piet A. van Rijn, Martin Beer, Polly Roy

**Affiliations:** a Department of Pathogen Molecular Biology, London School of Hygiene and Tropical Medicine, London, United Kingdom; b Institute of Diagnostic Virology, Friedrich-Loeffler-Institut, Greifswald, Insel Riems, Germany; c Université Paris-Est ANSES Alfort, UMR 1161 ANSES/INRA/ENVA, Maisons-Alfort, France; d Unit Vesicular and Exotic Diseases, CODA-CERVA, Uccle, Belgium; e Central Veterinary Institute of Wageningen University, Wageningen, The Netherlands; f Department of Biochemistry, North-West University, Potchefstroom, South Africa; University of Iowa

**Keywords:** Next-generation vaccine, bluetongue virus, reverse genetics

## Abstract

Bluetongue virus (BTV) is endemic in many parts of the world, often causing severe hemorrhagic disease in livestock. To date, at least 27 different serotypes have been recognized. Vaccination against all serotypes is necessary to protect susceptible animals and to prevent onward spread of the virus by insect vectors. In our previous studies, we generated replication-deficient (disabled infectious single-cycle [DISC]) virus strains for a number of serotypes and reported preliminary data on their protective efficacy in animals. In this report, to advance the DISC vaccines to the marketplace, we investigated different parameters of these DISC vaccines. First, we demonstrated the genetic stabilities of these vaccine strains and also the complementing cell line. Subsequently, the optimal storage conditions of vaccines, including additives, temperature, and desiccation, were determined and their protective efficacies in animals confirmed. Furthermore, to test if mixtures of different vaccine strains could be tolerated, we tested cocktails of DISC vaccines in combinations of three or six different serotypes in sheep and cattle, the two natural hosts of BTV. Groups of sheep vaccinated with a cocktail of six different vaccines were completely protected from challenge with individual virulent serotypes, both in early challenge and after 5 months of challenge without any clinical disease. There was no interference in protection between the different vaccines. Protection was also achieved in cattle with a mixture of three vaccine strains, albeit at a lesser level than in sheep. Our data support and validate the suitability of these virus strains as the next-generation vaccines for BTV.

**IMPORTANCE** Bluetongue (BT) is a debilitating and in many cases lethal disease that affects ruminants of economic importance. Classical vaccines that afford protection against bluetongue virus, the etiological agent, are not free from secondary and undesirable effects. A surge in new approaches to produce highly attenuated, safer vaccines was evident after the development of the BTV reverse-genetics system that allows the introduction of targeted mutations in the virus genome. We targeted an essential gene to develop disabled virus strains as vaccine candidates. The results presented in this report further substantiate our previous evidence and support the suitability of these virus strains as the next-generation BTV vaccines.

## INTRODUCTION

Since the development of the first bluetongue virus (BTV) reverse-genetics (RG) system in 2008 (1), there has been a surge of new candidate vaccines against BTV and related orbiviruses (2–6). The RG system has facilitated the creation of targeted mutant virus strains that elicit protective antibody responses but avoid the undesirable side effects of classical attenuated vaccines that had been generated by serial passages of the virus in tissue culture (reviewed by Dungu et al. [7]). The mutations associated with attenuation in these classical vaccines are unknown, and the degree of attenuation and transmission risk of any one vaccine strain is difficult to assess before its use in the field. The new targeted attenuated or replication-defective orbivirus vaccine strains are highly desirable, particularly for vaccines against BTV, the causative agent of vector-borne bluetongue disease in ruminant livestock. There are numerous BTV serotypes (up to 27 serotypes currently identified) that are endemic around the world, and classical live attenuated vaccines are marketed for many of these serotypes. Although these vaccines are generally protective, many vaccine strains do not afford complete protection and often have severe clinical and teratogenic effects in the fetus themselves (8, 9). Furthermore, there is very limited cross-reaction between BTV serotypes, and vaccination or infection with one serotype does not confer protection against other serotypes (10). More importantly, vaccination with these live virus vaccine strains can result in viremia sufficient for uptake and onward spread by Culicoides sp. vectors (11) leading to outbreaks (12) with devastating consequence for the farming industry. Potentially, this could lead to reversion to virulence and virulent reassortants (13). Furthermore, live virus vaccines are not distinguishable from the circulating viruses by serological methods alone (14). Differentiation between infected and vaccinated animals (DIVA) is important to control the spread of the disease.

The BTV particle is a nonenveloped double-capsid structure consisting of seven structural proteins (VP1 to VP7) and a genome of 10 double-stranded RNA (dsRNA) segments (15, 16). The inner capsid or core is made up of two major proteins, VP7 and VP3, and surrounds three enzymatic minor proteins, VP1 (polymerase), VP4 (capping enzyme), and VP6 (helicase and RNA packaging protein), in addition to the genomic dsRNAs (17, 18). The outer capsid is made up of two major proteins: the serotype-specific cellular attachment protein VP2 and the membrane penetration protein VP5 (19, 20). Core proteins are highly conserved across the different serotypes; however, the two outer capsid proteins are variable, particularly the receptor-binding protein VP2. Four nonstructural proteins (NS1 to NS4) are synthesized in virus-infected cells but are not part of the virion.

We have exploited the conserved nature of the core proteins, particularly the enzymatic VP6 protein, to generate a range of serotype-specific replication-deficient BTV (*viz*., disabled infectious single-cycle [DISC] virus vaccine strains) using RG systems. In previous reports, we have demonstrated the protective efficacy of a number of BTV DISC vaccines both in sheep and in cattle (2, 3). In current studies, we further characterize these vaccine strains and generate relevant information required for successful vaccine production. First, we improved the genetic stability of the DISC virus strains and determined the optimum storage conditions for the viability of DISC viruses at room temperature. Desiccation of the DISC vaccine strain in the presence of suitable additives preserved complete protective immunity of the vaccine in sheep. Furthermore, we demonstrated that protection afforded by DISC vaccine in a single or cocktail format was effective even as early as 21 days postvaccination (dpv) or as late as over 5 months in vaccinated sheep. Cocktail vaccines were shown to elicit protective immunity in cattle, albeit at a lesser level than that in sheep at the same dosage. More importantly, there was no evidence of interference in vaccinated sheep between the serotypes included in the cocktail vaccine. Overall these data highlight the RG as a very strong platform to tailor an efficacious and rapid response to outbreaks involving one or more BTV serotypes.

## RESULTS

### Genetic stability of BTV replication-deficient virus and the complementing cell line.

**FIG 1:**
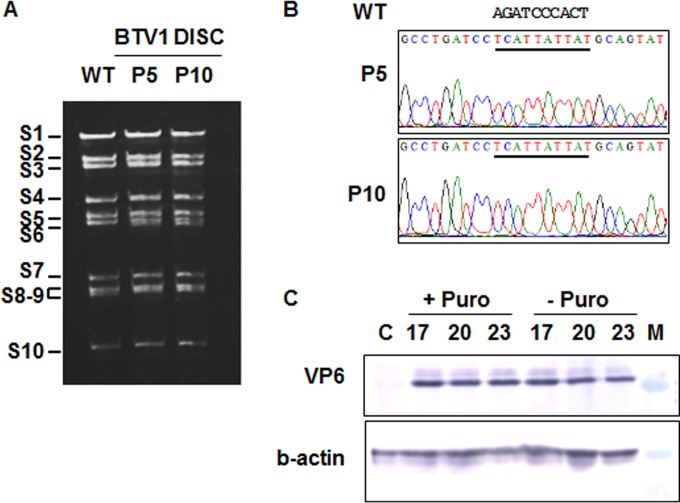
Stability analysis of the vaccine virus strain and complementing cell line. (A) Genomic RNA profile of the BTV1 DISC strain in complementing BS9 cells at passages 5 (P5) and 10 (P10). WT, BTV1 control. Positions of RNA segments (S1 to S10) are indicated. (B) Sequence electropherograms of S9 RT-PCR products from passages 5 and 10. Mutations in S9 are underlined, and the sequence of the WT virus is shown. (C) Expression of VP6 in the complementing BS9 cell line in the presence (+ Puro) or absence (− Puro) of puromycin at passages 17, 20, and 23 by Western blotting. Lane C, normal BSR cells; lane M, protein marker.

To ensure the genetic stability of replication-deficient viruses (DISC strains), several stop codons were introduced in the segment S9 that disrupt the expression of VP6 without affecting the length or packaging signals of the S9. The rationale was to minimize the impact of the changes on the segment's stability and to reduce any pressure for reversion after several passages (more than 5 passages in complementing cells). BTV1 DISC virus was recovered in complementing cells, and its genome profile was analyzed. Gel electrophoresis of the purified genomic dsRNAs of passage 5 and 10 viruses had identical profiles that are comparable to that of the wild type (WT) (Fig. 1A). Furthermore, the analysis of sequences of the segment S9 (VP6) found no difference between the passage 5 and 10 viruses, and there were no unexpected changes in the sequence of the mutant S9 segment (Fig. 1B). These data suggested that these DISC viruses are stable after multiple passages.

To ensure further that no reversion occurred during the generation of virus stocks, viruses grown in complementing BS6 cells at passage 5 or 10 were passaged 3 additional times on noncomplementing BSR cells and in insect Culicoides cells (KC cells). Infection in cells was monitored for cytopathic effect, the presence of genomic RNA by reverse transcription (RT)-PCR, and infectious virus progeny by plaque assay. No replication was detected in any case (data not shown), supporting that the DISC strain is genetically stable and there is very little possibility, if any, for reversion to infectious virus.

The stability of VP6 expression in the BS9 complementing cell line was also tested. In previous reports, the BSR VP6 cell line was used at very low passage number, and the stability and the level of expression of VP6 after higher passages have not been assessed (2). Even after 23 passages, the BS9 cells were still expressing VP6 at similar levels compared to earlier passages (Fig. 1C), indicating that the level of expression was not affected by serial passages. Furthermore, this cell line was maintained in selective medium containing puromycin, the selection reagent, but for vaccination purposes, antibiotics should be removed from the preparation. Therefore, we investigated if the cell line can be kept without puromycin with minimum effect on VP6 protein expression. The expression dependency of VP6 on the continuous presence of the puromycin was not evident in this study as after several passages in medium without puromycin, the level of VP6 remained stable (Fig. 1B). These results suggest that viable vaccine candidates can be prepared in a BTV VP6 cell line in the absence of antibiotics.

### Effects of different sugars and temperature on BTV1 DISC virus stability.

**FIG 2:**
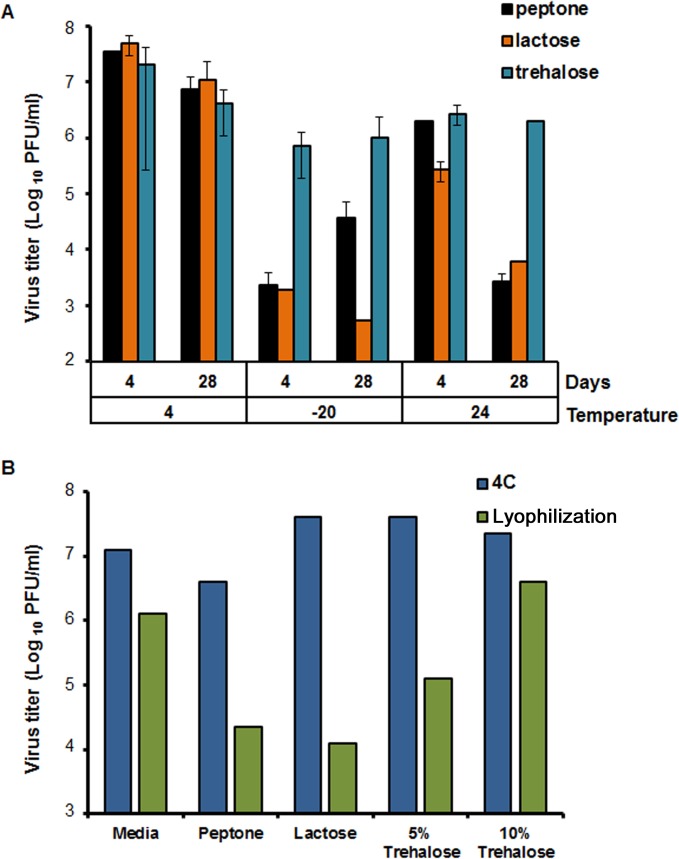
Optimization of storage conditions for the BTV1 DISC virus. (A) Virus titers in the presence of peptone, lactose, or trehalose at the indicated temperature (4°C, −20°C, or 24°C) at 4 and 28 days. Titers were expressed as PFU per milliliter and plotted in logarithmic scale. (B) Virus titers after desiccation in the presence of peptone, lactose, or two concentrations of trehalose (5% and 10%). No additive (PBS) and 4°C storage were included for comparison.

To investigate the effect of storage conditions and the incorporation of additives on maintaining vaccine virus titer, a virus stock was divided into 5 groups, and different protective agents, such as peptone, lactose, or 5% or 10% trehalose were added. Furthermore, since storage temperature is also critical, the ability of the different formulations to maintain particle infectivity was tested at a range of temperatures, from −20°C and +4°C to room temperature (24°C). Stocks were stored for either 4 or 28 days prior to assessing the virus titer. Furthermore, since a low pH in the environment causes the virus to shed the outer capsid layers, pH was also monitored to ensure that treatment and storage conditions were not unduly influencing virus stability. Storage of the vaccine preparation at 4°C with any of the 3 additives was identified as the best condition, with only an ∼1-log_10_ drop in titer from day 4 to day 28 (Fig. 2). Storage of the virus samples at −20°C resulted in a significant drop in virus titer (about 5 log_10_) in the presence of peptone and lactose but less so in the presence of trehalose. Although there was a decrease in virus titer (about 1 log_10_) after the addition of trehalose at all temperatures, there was no significant difference between the day 4 and day 28 samples, with titer remaining consistent at all temperature tested. For all conditions, the pH ranged from 7.5 to 8.5 with no significant variations. These results suggested that the addition of trehalose to the vaccine strain maintained the infectivity up to 28 days at all temperatures tested.

Elimination of the cold chain is an important improvement in delivering a vaccine to animals in the field and minimizing the cost associated with production and storage. The effect of addition of different stabilizing reagents (peptone, lactose, or trehalose) followed by desiccation on DISC vaccine titer was analyzed and compared to that of a nondesiccated preparation kept at 4°C (Fig. 2). The addition of these additives did not affect the virus infectivity, with titers similar to those of the control (media only) stock virus (1 × 10^7^ to 5 × 10^7^ 50% tissue culture infective doses [TCID_50_]/ml). This result was consistent with initial screening results (Fig. 2). The storage at 4°C with peptone alone resulted in a titer decrease from 1 × 10^7^ to 5 × 10^7^ TCID_50_/ml to 1 × 10^6^ TCID_50_/ml. The effect of desiccation was also assessed and resulted in a decrease of 0.6 to 4 log_10_ in virus titer in all samples. The addition of 10% trehalose to the media maintained the infectivity of the virus in comparison to those of peptone, lactose, and 5% trehalose. The virus titers decreased from a range of 1 × 10^7^ to 5 × 10^7^ TCID_50_/ml to a range of 1 × 10^4^ to 1 × 10^5^ TCID_50_/ml. The addition of 10% trehalose to virus stocks prior to desiccation provided the best means to maintain infectivity. Desiccated stocks kept at room temperature or 4°C for up to 8 weeks also appeared to maintain virus titer.

### Influence of vaccine desiccation on immunological response and protection to BTV1 challenge.

**FIG 3:**
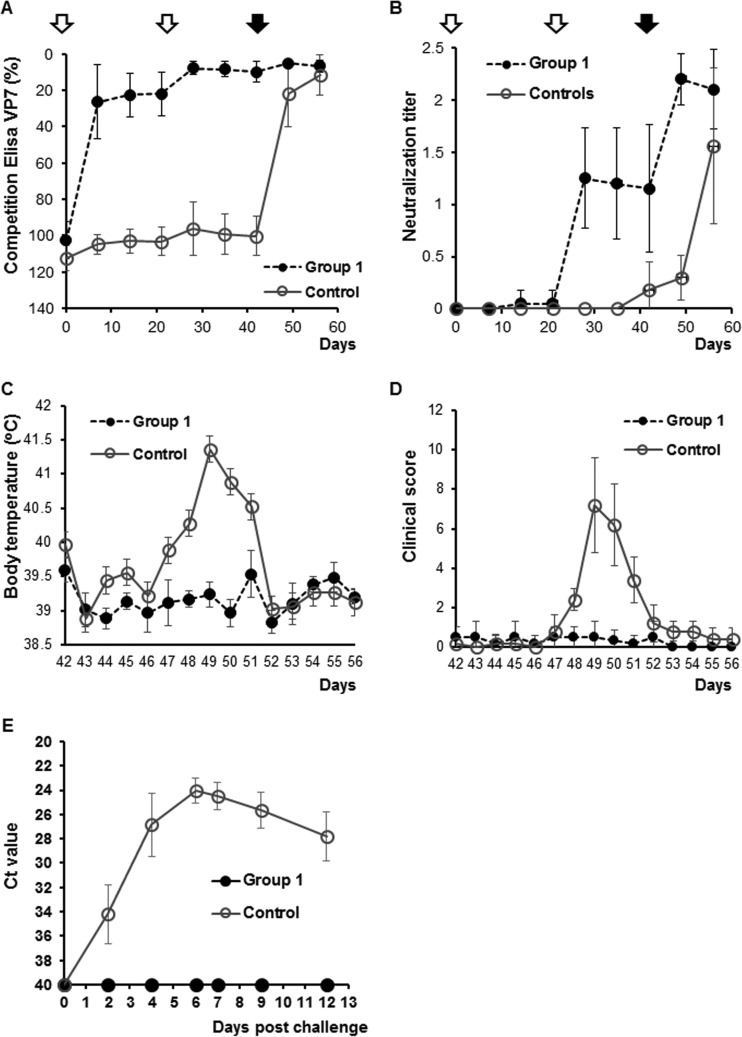
Immune response and protection afforded by BTV1 DISC vaccine in sheep. The immune response to the vaccine was monitored by VP7 group-specific competitive ELISA (A) and seroneutralization test (B) at different time points. Animals were inoculated twice (empty arrows) with BTV1 DISC vaccine at days 0 and 21 (group 1). The control group was inoculated with cell lysate. All animals were challenged at day 42 (full arrow). (C) Body temperature (^o^C) and (D) clinical scores were recorded daily for the vaccinated (group 1) or nonvaccinated (control) animals. (E) Detection of BTV genome in blood after challenge by qRT-PCR. A *C_T_* value lower than 40 was considered positive for BTV replication.

The effect of trehalose and desiccation on neutralizing antibody responses in sheep was also examined. A group of 6 sheep was vaccinated with the desiccated DISC virus using the standard two-dose regimen of 21 days apart (at days 0 and 21). A control group of 6 animals was vaccinated with only phosphate-buffered saline (PBS) as described previously (21). Development of the BTV antibody response in sheep was monitored by a VP7 group-specific antigen enzyme-linked immunosorbent assay (ELISA). As expected, sheep in the control group did not trigger any antibody response to VP7. However, all vaccinated animals were seroconverted. Sera of all vaccinated animals had antibodies that recognized VP7 by day 7 after the first vaccination and remained positive until the end of the experiment. The data indicated that the desiccation or trehalose did not alter the conformation of VP7 of the DISC virus and possibly the overall biological property of the DISC virus strain remained intact (Fig. 3A). To further investigate if the VP2 structure was not disrupted by desiccation and that the vaccinated sheep still elicited neutralizing antibodies against virulent BTV1 virus, each sheep serum was assessed for virus neutralization in tissue culture. All animals inoculated with the desiccated vaccine in a prime-boost regimen had detectable neutralizing antibodies (seroneutralization titers of 8 to 128) after the second inoculation (Fig. 3B).

Both control and vaccinated animal groups were challenged with virulent BTV1 on day 42. The vaccinated sheep did not develop fever or display any clinical symptoms of BT. However, all control sheep had fever for 5 days after challenge, with a peak at 7 days postchallenge (dpc) (Fig. 3C). These animals also showed classical clinical signs of BT (Fig. 3D), including increased rate of breathing, mild depression, decrease in appetite, and conjunctivitis after challenge with virulent BTV1.

Serological analysis demonstrated that all postchallenged sheep elicited a response to the BTV group-specific antigen VP7, by ELISA (Fig. 3A). Furthermore, neutralizing antibodies against BTV1 were detected in all animals (Fig. 3B). Prior to challenge, vaccinated animals had detectable SN titers, which were still maintained until the end of the experiment. However, in the control group, neutralization titers were detectable only after the challenge.

To ensure that the vaccine was not only protecting against disease but was completely efficacious in preventing virus replication, blood samples of each animal were examined for circulating viral genome by reverse-transcription quantitative PCR (RT-qPCR). None of the animals inoculated with the DISC vaccine had any detectable circulating BTV1 (Fig. 3E). In comparison, BTV1 genome was detected in all of the control animals. A typical viremic response was observed for the control group: peak viremia occurred at day 6 followed by a slow reduction of the virus in the circulating blood (Fig. 3E).

### Early protection afforded by a MultiDISC (BTV1, -2, -4, -8, -13, and -21) vaccine trial in sheep.

Our previous study demonstrated that a vaccine cocktail of six DISC serotypes (BTV1, -2, -4, -8, -13, and -21) induced neutralizing antibodies and afforded protection against a virulent BTV challenge (3). To investigate if this protection can be afforded early after vaccination, two groups of six sheep were vaccinated with the multivalent vaccine (MultiDISC) and challenged 21 days after the vaccination with either BTV2 or BTV8 serotypes.

**FIG 4:**
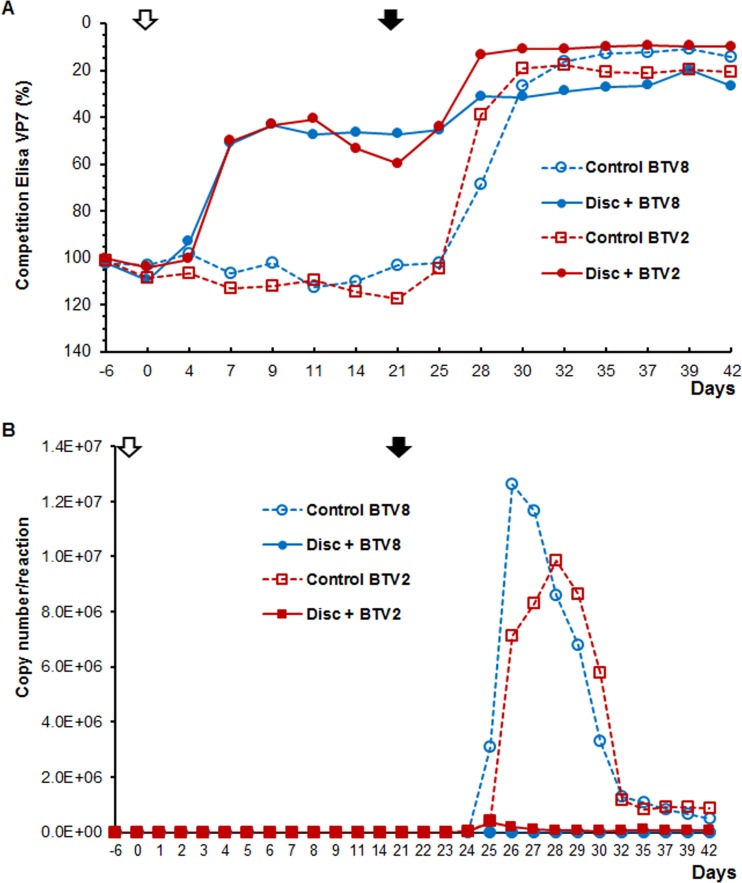
Early protection in sheep vaccinated with a MultiDISC vaccine. (A) Seroconversion of vaccinated and control animals by group-specific VP7 competition ELISA. All animals were vaccinated once (open arrow) and challenged at 21 dpv (solid arrow). (B) Virus load in blood was tested by qRT-PCR and calculated as the average number of copies per reaction per group.

The immune response to the vaccine was evident as all animals were seroconverted as early as 7 dpv, as shown in the competitive ELISA (cELISA) (Fig. 4A). While progressive decrease in the titer of VP7 antibodies was observed until the day of the challenge (day 21) with the early-detection ELISA, the titers remained stable with the cELISA. At the challenge day, animals were still positive. However, the seroneutralization (SN) response of vaccinated animals was much weaker. BTV neutralization was undetectable in almost all vaccinated animals, except in only one sheep, which had a titer of 16 to 32 for BTV1 and BTV4 (data not shown).

All animals in the vaccinated groups showed protection from BTV infection, albeit at different levels by a diagnostic pan-BTV real-time RT-PCR in blood samples (Fig. 4B). The genomic copy number detected indicates that in the BTV8-challenged group, 3 out of 4 animals remained negative, with the exception of borderline RNA detections after challenge (1 to 4 dpc), probably due to the detection of the inoculated challenge virus. The 4th animal became positive at 3dpc, with a maximum at 4 dpc, and copy numbers never exceeded 2 × 10^3^ copies/reaction (c/r). Subsequent samples remained positive until 19 dpc, although with decreasing copy number. In comparison, the mean maximum copy number in the control animals infected with BTV8 was 1.26 × 10^7^ c/r. These results indicate that although the immunity is not 100% sterile in all of the vaccinated and subsequently challenged animals, the viremia seen in the one animal was significantly lower (almost 4 log units) than that of the control animals (Fig. 4B).

In the BTV2-vaccinated group, all animals became PCR positive after challenge. The copy number increased to a maximum mean of 5 × 10^5^ c/r. Nevertheless, this value was significantly lower than the mean maximum of 1.25 × 10^7^ c/r in the BTV2-infected control animals, demonstrating the important effect of the vaccine. Altogether, the data indicate that the replication-deficient virus has a protective effect on vaccinated animals even a few days after inoculation.

### Duration of immunity of MultiDISC vaccine in sheep.

Understanding the long-lasting protection afforded using this vaccine is essential to control an outbreak and prevent spreading of the disease. For BTV immunity, we analyzed the duration of the protection afforded with the MultiDISC vaccine, including the same serotypes tested previously. The immunization scheme included two doses, 21 days apart, and a challenge with a virulent strain (either BTV2 or BTV8) after 154 days.

**FIG 5:**
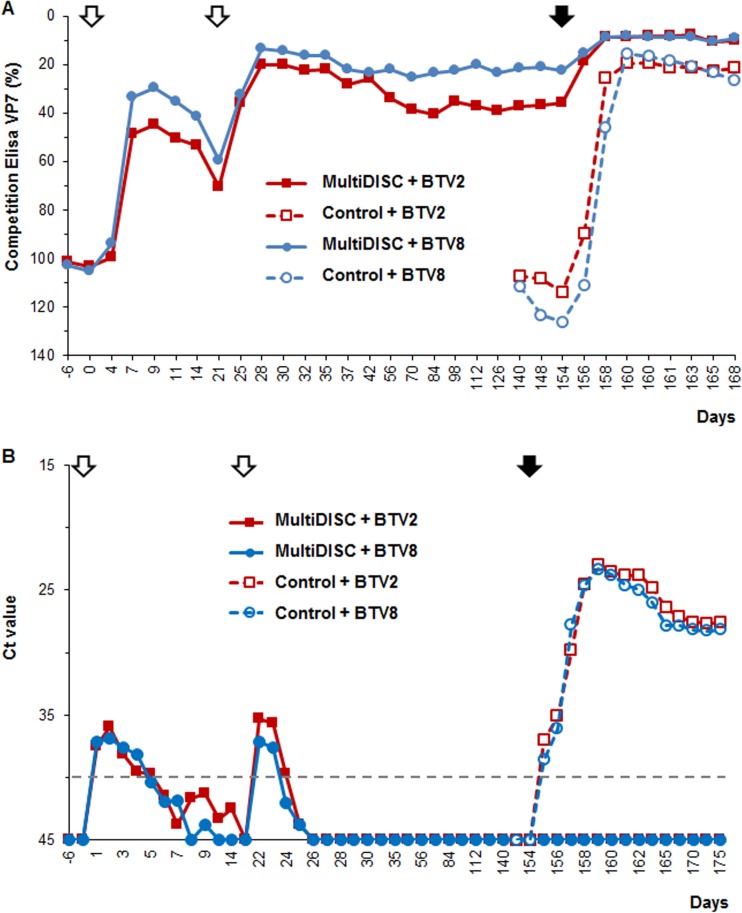
Long protection test in sheep vaccinated with a MultiDISC vaccine. (A) Detection of group-specific antibody response (against BTV VP7) using competition ELISA. Animals were vaccinated twice 21 days apart (empty arrows) and challenged 154 dpv (full arrow). (B) Virus load was determined by RT-PCR, and the results were expressed as mean *C_T_* values. The dashed line represents the PCR cutoff; results below the line are considered negative.

All vaccinated animals seroconverted at 7 dpv, and a clear booster effect was seen in all animals after a second dose with high titers until challenge at 154 dpv (Fig. 5A). Following challenge, the immune response titer remained high and stable until the end of the experiment. All control animals were negative at the start of the test, but clinical signs were evident 5 dpc with a virulent strain (data not shown) and seroconverted at 7 dpc (Fig. 5A).

Presence of BTV RNA was monitored by pan-BTV diagnostic real-time RT-PCR in blood samples and expressed as average cycle threshold (*C_T_*) per group (Fig. 5B). Following challenge with either BTV2 or BTV8 strains at 154 dpv, no indication of virus replication was observed for the vaccinated/challenged animals, and this remained so until the end of the experiment, suggesting complete protection. Between 1 and 3 dpc, all control animals became viremic with very high genomic copy numbers suggesting BTV replication in these unprotected animals. Although the genome derived from the DISC strain was detected after each vaccination, all animals were negative on the challenge day, supporting that the vaccine was not able to replicate in the host.

**FIG 6:**
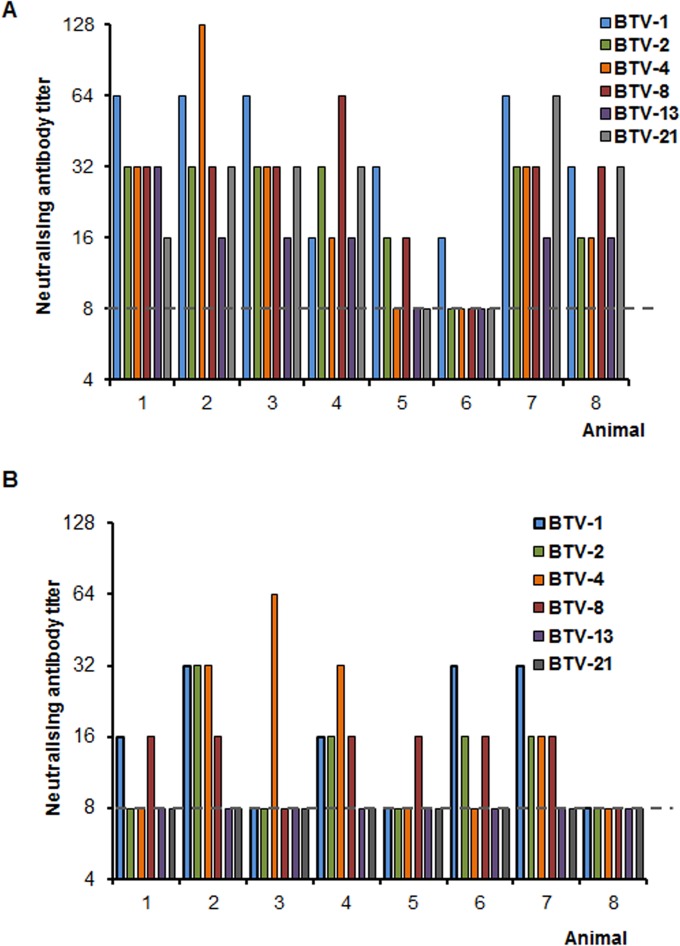
Long-term neutralization activity in sheep inoculated with a MultiDISC vaccine. Neutralizing titer was tested against all serotypes included in the cocktail vaccine at two time points: 42 (A) and 154 (B) days postvaccination. Titers below the threshold (dashed line) were considered negative.

The nature of the immune response in this long-duration experiment was further analyzed by determining the neutralization activity in the sera of vaccinated animals. Two time points—21 days after the boost (42 dpv) and at 154 dpv, the day of the challenge—were tested for neutralization activity against all serotypes included in the vaccine cocktail. At 42 pdv, all animals presented some level of neutralization against at least one serotype in the vaccine (Fig. 6A). Two animals responded poorly, and one of them showed reactivity only against BTV1. As expected, the long-term neutralization activity was lower at 154 dpv (Fig. 6B). Seven animals showed some neutralization activity against at least one serotype, and only one animal showed no significant neutralization.

### Evaluation of TriDISC vaccination (BTV2, -4, and -8) in cattle.

In a previous report, we showed that cattle vaccinated with monoserotype DISC virus BTV2, BTV4, or BTV8 were able to elicit a protective neutralizing antibody response (3). To investigate the effectiveness of a cocktail vaccine containing three BTV serotypes (BTV2, -4 and -8 [TriDISC]) in cattle, two independent trials were undertaken. In the first trial, 15 animals were vaccinated, and 9 were used as controls. In the second trial, 12 animals were vaccinated, while 6 were used as controls. In both trials, animals were inoculated twice with the TriDISC cocktail and challenged with BTV2, BTV4, or BTV8 virulent strains.

**FIG 7:**
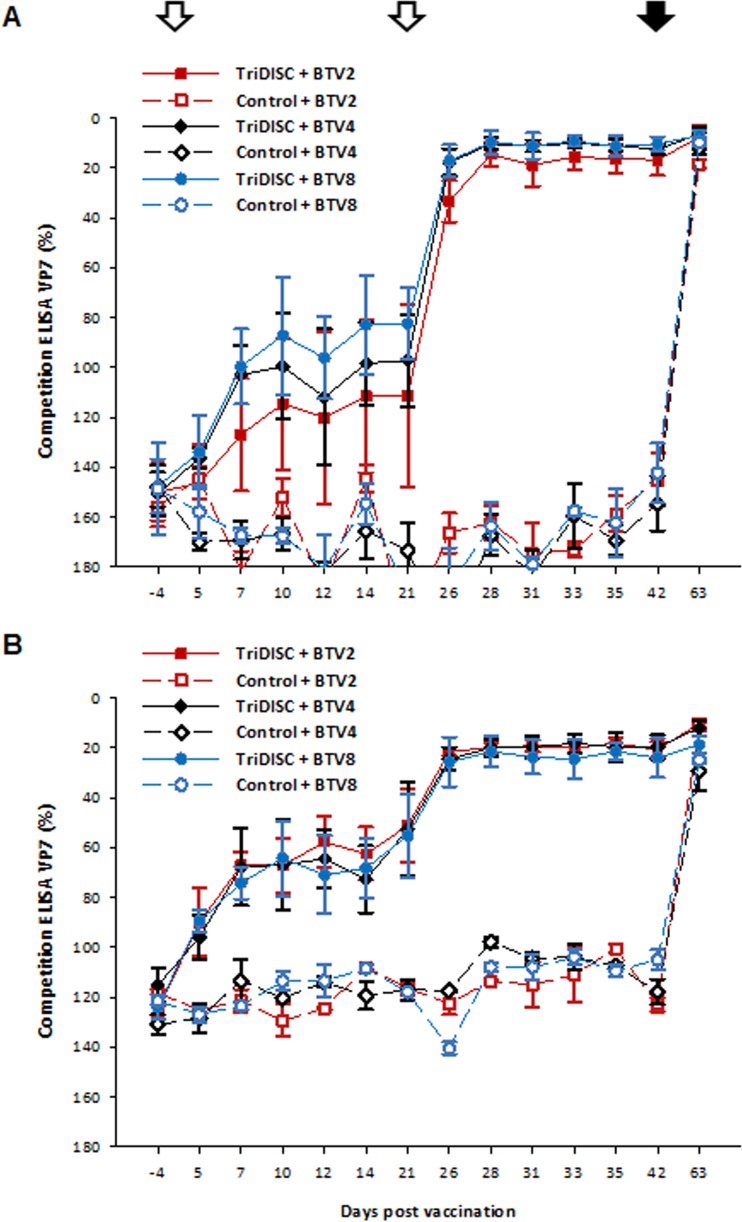
Immune response in cattle inoculated with a TriDISC cocktail vaccine. Two trials were performed (A and B). Animals were vaccinated twice 21 days apart (open arrows) and challenged at 42 dpv (solid arrow). Seroconversion was monitored by a BTV serogroup-specific ELISA. For each group, the mean and 95% confidence interval are shown.

A pronounced immune response was detected in all cattle vaccinated with the TriDISC cocktail after boost vaccination by group-specific cELISA in both trials (Fig. 7A and B) and remained positive until challenge, indicating all animals were seroconverted.

The immune response was further analyzed by determining the neutralization titer by seroneutralization (SN) assay for the animals on the challenge day. The SN titer in the first trial showed a weak response (∼8), with only one animal with a detectable titer (∼16) against BTV4. Because a weak neutralization titer was detected in the first trial, in the second trial, a higher dose of DISC virus was used to improve the immune response. In the second trial, the response was more promising. On the challenge day, 67% of the vaccinated animals (8 out of 12) had neutralizing titers between 16 to 64 against BTV8. No SN titer was detected for BTV2 and BTV4 included in the vaccine. None of the control animals showed any neutralizing antibody response on the challenge day.

**FIG 8:**
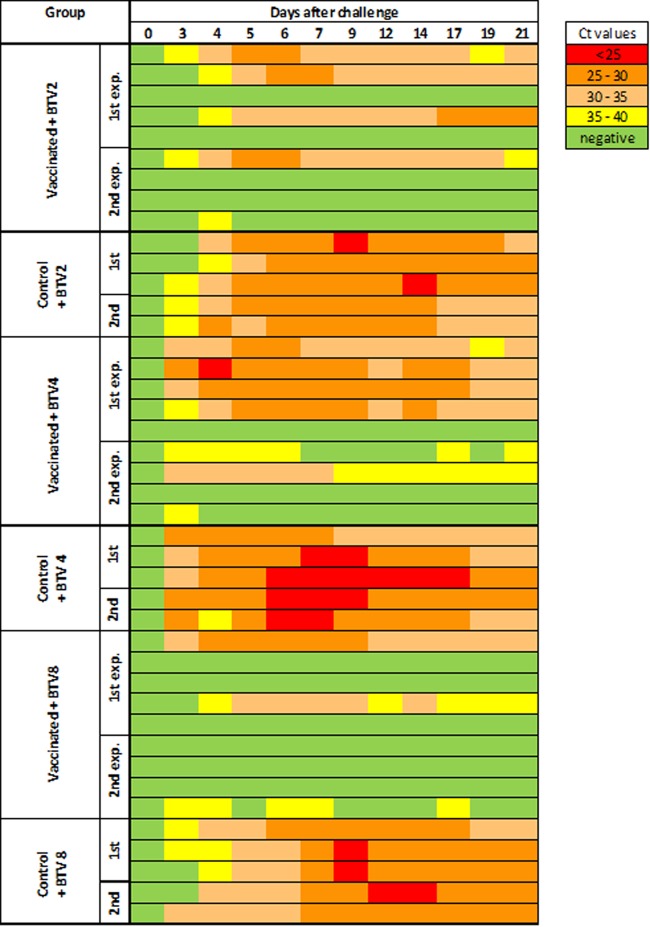
Viral load in cattle inoculated with a TriDISC cocktail vaccine after challenge with virulent BTV. BTV genomic RNA content in serum was measured by real-time RT-PCR. Animals were vaccinated with the cocktail vaccine and challenged with BTV2 or BTV4 or BTV8 as indicated. Low to high loads (high to low *C_T_* values) are represented by a color gradient from green to red.

After challenge with virulent virus, viral genome was detected by RT-qPCR in the blood of all animals in the three control groups on 3 or 4 dpc with high copy numbers (Fig. 8). In the BTV2 challenge group of the first trials, 3 out of 5 vaccinated animals were positive for more than 1 day, compared to the second trial group, where 75% of animals were protected as only 1 out of 4 was positive. Similar results were obtained with BTV4-challenged groups, with 4 out of 5 positive in the first trial group and 2 out of 4 in the second trial group, consistent with the weak neutralizing antibody response or lack of response in these animals.

In contrast, animals challenged with BTV8 were better protected, as 3 out of 5 (first trial) and 3 out of 4 (second trial) showed no virus replication.

In addition to the prevention of viremia, in 14 of 27 (combined first and second trials) animals (52%), a clear effect of the vaccination was also seen in viremic animals in the vaccination groups. Their viral loads were significantly reduced compared to those in the controls. In the BTV2 challenge group, the mean *C_T_* value among PCR-positive samples from vaccinated animals was 32.3 ± 2.6, compared to 28.8 ± 3.05 in the control animals, for BTV4, it was 31.7 ± 4.1 versus 27.8 ± 3.2, and for BTV8, it was 33.3 ± 4.5 versus 28.9 ± 3.7 (Fig. 8). No obvious clinical signs attributable to BTV infection were observed in the TriDISC-vaccinated cattle. In control animals, on the other hand, clinical signs included nasal and ocular discharge and respiratory distress, as well as muco-cutaneous lesions around the nose and mouth, with overall clinical scores between mild and moderate.

## DISCUSSION

In recent years, a number of new types of vaccine candidates for BTV have been developed using reverse genetics of BTV. Although several of these candidate vaccines have been shown to elicit protective immunity in sheep, none have been commercialized to date. In this report, we investigated a number of essential vaccine parameters of our replication-deficient DISC virus strains to forward these candidate vaccines to the marketplace. One of the most important criteria is the genetic stability of these vaccine strains, which were initially designed to exclude an essential gene of the viral genome replication by deleting the VP6 coding region of segment S9 altogether (2). Since large deletions often compromise genetic stability, we generated an alternate VP6-deficient virus in which multiple stop codons were introduced into the gene. We found that this strategy has all the benefits of the VP6 deletion mutant viruses that we reported previously but superior genetic stability compared to the previously reported construct (2). There was no evidence of genetic pressure for reversion after multiple serial passages. Furthermore, for the first time, the optimization of the storage conditions of this vaccine strain was undertaken in the presence of stabilizing reagents followed by desiccation at room temperature. These conditions were shown to have minimal impact on vaccine virus titer. Moreover, these desiccated DISC vaccines were able to induce immune responses in a typical double-dose protocol 21 days apart that conferred complete protection in vaccinated sheep against virulent virus challenge, suggesting vaccine efficacy was not compromised by desiccation at room temperature. Additionally, and in compliance with the manufacturing requirements of vaccine formulations without antibiotics, we demonstrated that the complementing VP6 cell line maintains a constant level of VP6 expression even at high passage numbers and in the absence of the selective agent (puromycin) in the growth media. Altogether, our data showed that the DISC vaccine strain is genetically stable, and its virus titer can be maintained by desiccation in the presence of stabilizing reagents. These features are essential for mass production and vaccine administration in the field as no cold chain for delivery and handling is needed.

During an outbreak of BT disease, it is essential that the vaccination strategy respond fast and efficiently to the threat affording protection as early as possible to minimize the risk of infection and spread. In a previous report, we demonstrated that a single dose of a DISC virus strain was sufficient to confer complete protection against a challenge in sheep 42 days after vaccination (2). In the present study, we further demonstrated that a single dose of the 6 different serotypes in a cocktail could also protect animals as early as 21 days after vaccination as no viremia was detected in vaccinated and challenged sheep. Furthermore, previously we reported that a cocktail of six different DISC virus strains representing six BTV serotypes was completely protective in sheep against the virulent strains that were tested, indicating the possibility of generating a mixture of vaccine strains without any interference between different serotypes (3). However, the vaccinated animals were challenged at 42 days after vaccination. Here, we used the same combination of cocktail and demonstrated the long-term protective efficacy of this cocktail by challenging the animals after 154 days of vaccination. Overall these results suggest that even when the neutralization activity in sera was very low *in vitro*, the immune response of the sheep afforded by this MultiDISC vaccine was sufficient to confer protection against representative virulent strains included in the cocktail even more than 3 months after vaccination.

Virus-like particles (VLPs) of BTV had also been shown to afford long-term (up to 14 months) protection in sheep (22). A similarly inactivated vaccine (23) and a live attenuated (24) vaccine have also been shown to elicit immune responses 1 year after vaccination. Therefore, it is likely that DISC vaccines could also sustain their immune responses in animals for a long duration. However, further studies will be needed.

Supporting the potential of these next-generation vaccines as candidates for BTV control, in recent years, RG-based strains bearing mutations that abolish the expression of one of the nonstructural proteins (NS3/NS3A) have been developed (5). These disabled infectious single-animal (DISA) vaccines are different from our strains because they are highly attenuated live virus strains, as these viruses are capable of replication, although at a very low level. Current live attenuated BTV vaccines that are used in the field are not completely safe as some are linked with undesirable secondary reactions, such as teratogenic effects, reversion to virulence, and the risks of transmission to vectors. Vaccine studies with these DISA vaccines showed protection in vaccinated animals and inability to replicate in the Culicoides vector (25). The vaccine candidates that we are presenting in this report do not replicate either in cell culture or in the susceptible hosts and in that respect are more similar to an inactivated vaccine, but with several advantages. No inactivation process that increases the production cost is necessary with the DISC vaccines, making these vaccines more cost-effective. More importantly, since DISC strains cannot complete a replication cycle in normal cells or in the bloodstream of a vaccinated host, DISC strains are present only for a short period, thus minimizing the risk of transmission to insect vector, reassortment, or reversion to virulence. However, these defective strains are capable of entering the cells and expressing viral proteins (2) that are available for the host immune system for an improved response.

Results for the efficacy of the candidate vaccines were slightly different between the two hosts, cattle and sheep. The results of the competitive ELISA revealed a weak humoral immune response after the first dose of the TriDISC vaccination in cattle.

The good antibody response after the second dose suggests that double doses of DISC cocktail vaccines are necessary for cattle, at least with the dosages tested, different from the outcome of the sheep vaccination trials. Nevertheless, a majority of cattle vaccinated with the TriDISC cocktail vaccine in the second trial were completely protected against virulent challenge. Since the SN titer was very low in cattle, no clear correlation between the neutralizing response and the viremia could be established, and further studies are needed to determine other factors involved in the protection (i.e., T cell response). Although the first trial group had a lower titer of DISC vaccines than the second group and some animals had low levels of BTV replication, overall, the TriDISC vaccination induced a significant reduction of the BTV viral load in peripheral blood and protected against clinical disease in all animals. Furthermore, it is known that a reduction of viremia also markedly reduces the risk of transmission by Culicoides midges (11). Therefore, the TriDISC vaccine would most likely have a strong impact not only on the clinical outcome but also on the virus spread.

It is noteworthy that sheep could be protected by inactivated (26) or DISC (2) vaccines with a single-shot application, while cattle generally needed two applications for complete protection (27). In addition, the observed pattern of neutralizing activity could indicate interference between the serotypes included as reported for live attenuated vaccine strains (28, 29). This possible interaction should be further studied, and the role of the strains' differences and virus titers should be investigated. Different strain combinations or higher titers for particular strains in cocktail vaccines need to be tested in order to improve protection. Additionally, commercial and some experimental vaccines include in their formulation adjuvants such as aluminum hydroxide, Montanide ISA 206 (Seppic), or immunostimulating complex (ISCOM) that increase the immune response (30–32). Adjuvants were not included in this trial but can be beneficial in the formulation of the replication-deficient cocktail vaccines for cattle, a possibility that needs to be explored.

It is noteworthy that in the early-detection and long-duration trials in sheep presented in this report, the genome of the vaccine strain was detected in the blood for only a short period and at a very low levels after each inoculation. Since genomes of the vaccine strains are deficient of replication, there is no possibility for reassortment between the vaccine strains and infecting virus strains.

Replication-deficient vaccine strains are promising candidates for commercial development to control BTV. Research into the mechanisms involved in the response to these vaccines will help to develop a platform for a rapid response to a potential outbreak with single or multiple serotypes.

## MATERIALS AND METHODS

### Cell lines and viruses.

BSR cells (a BHK-21 subclone) were maintained in Dulbecco modified Eagle medium (DMEM [Sigma-Aldrich]) supplemented with 5% (vol/vol) fetal bovine serum (FBS [Invitrogen]). The stable BSR-VP6 cell line BS9 was grown in DMEM supplemented with 5% FBS and 7.5 μg/ml of puromycin (Sigma-Aldrich) as previously described (2, 21). Parental BTV and DISC virus stocks were obtained by infecting BSR or BS9 cells, respectively (2, 3). Titration of all deficient viruses was performed in the BS9 cell line, and the virus titer was determined by plaque assay (PFU) or endpoint titration (50% tissue culture infective dose [TCID_50_]) as described previously (33).

### Recovery of replication-deficient BTV1 DISC virus.

A triple-stop-codon mutation at residues 87 to 89 in VP6 was introduced into BTV10 S9 by site-directed mutagenesis (34) using the T7 plasmid pUCT710S9 (2) and the following primers: 10S9TS257-F (5′-GACGCATACATACTGCATAATAATGAGGATCAGGCACAAAAGG-3′) and 10S9TS257-R (5′-CCTTTTGTGCCTGATCCTCATTATTATGCAGTATGTATGCGTC-3′). For virus recovery, the BTV reverse-genetics system was used (2).

### Stability of the vaccine strain BTV1 DISC and the stable cell line BS9.

The genetic stability of the replication-deficient virus was tested by serial passage of the virus in the complementing cell line BS9. After 5 and 10 passages, the genomic dsRNA was extracted and purified using Tri reagent (Sigma-Aldrich) and analyzed by 11% PAGE in the presence of ethidium bromide.

The level of VP6 expression in the complementing BS9 cell line was assessed after serial passages in media in the presence or absence of puromycin. Cell lysates were analyzed at passage numbers 17, 20, and 23, and the expression of VP6 was detected by Western blotting using a specific antibody against BTV VP6. As loading control, the cellular protein β-actin was detected using a monoclonal anti-β-actin antibody (Sigma-Aldrich).

Virus stability at different storage conditions was tested by the addition of protective agents to the deficient virus stock BTV1 DISC. Three different additives were used for the stability test: peptone (1% Bacto peptone in phosphate-buffered saline [PBS]), lactose (1% lactose, 1% Bacto peptone in PBS), or trehalose to a final concentration of 5% or 10% prepared in PBS. Each additive was added to 1-ml aliquots of the vaccine sample and stored at −20°C, 4°C, or room temperature (24°C). Duplicate samples were taken at 4 and 28 days, and their infectivity was determined by titration.

For the desiccation test, additives were added to each sample and subjected to desiccation using a SpeedVac concentrator (Savant) until completely dry. The following day, the vaccines were reconstituted and the titer monitored. As a control, nondesiccated aliquots were stored at 4°C.

### Monoserotype vaccination with BTV1 DISC in sheep.

To assess the efficacy of the BTV1 DISC vaccine candidate after addition of trehalose (10%) and desiccation, 10-month-old healthy male and female sheep (Crossbred Pre Alps) were used. The animals were free of respiratory, digestive, umbilical, parasitic, and osteo-articular diseases and were seronegative for BTV by competitive ELISA test. Two groups of 4 to 6 animals were segregated at random. Group 1 was vaccinated twice with the vaccine (1 × 10^7^ TCID_50_) by the subcutaneous route on the lateral side of the thorax, 21 days apart. Group 2 was inoculated with a BTV-free BS9 cell lysate and was used as control. On day 42, all animals were challenged with BTV1 (4110. 13.06 BTV1 KC2), which was isolated from blood of an infected bovine in 2013 in Corsica (35), followed by passaging in KC cells twice. Several intradermal injections on the inner side of the right thigh were given. Clinical data were recorded as described previously (36). At the end of the experiment, all animals were sacrificed. Animals were treated according to the ethical rules of national and European regulations on animal welfare. Whole blood and sera were collected routinely (as indicated) to monitor the antibody response to BTV antigens and virus load.

### MultiDISC (BTV1, -2, -4, -8, -13, -21) vaccination trials in sheep.

All sheep experiments were performed under the guidelines of the European Community and were approved by the Committee on the Ethics of Animal Experiments of the Central Veterinary Institute (permits 2013.015 and 2013.016). Female Blessumer sheep (6 to 24 months old) were obtained from a Dutch farm and were free of BTV and BTV antibodies. Sheep were allocated randomly to groups of four animals at 1 week before the start of the experiment.

### Early protection.

Sheep of two groups were vaccinated with 1 ml MultiDISC vaccine (between 5 × 10^6^ and 4 × 10^7^ PFU/serotype). Two groups were mock vaccinated with BTV-free cell lysate. Sheep were injected subcutaneously (s.c.) over the back between the shoulder blades on both sides of the spinal cord. At 21 dpv, one vaccinated group and one nonvaccinated group were s.c. injected in 4 different places with 10^5^ TCID_50_ of BTV8/net07 (isolated on eggs and passaged three times on BHK-21 cells [BTV8/net07/e1/bhkp3]) or BTV-2/SAD2001/01 (isolated from sheep at the Pirbright Institute, United Kingdom, and grown once in embryonated chicken eggs, with two passages on BHK-21 cells and three passages on KC cells [BTV2/SAD01/01/e1/bhkp2/kcp3]) and used in a previous report (3). Samples of serum and EDTA-treated blood were collected at indicated days of the experiment. Clinical signs were scored as previously described (36).

### Duration of immunity.

Sheep of two groups (8 animals per group) were vaccinated with 1 ml of MultiDISC vaccine as described for early protection. At 21 dpv, animals received a booster vaccination. At 154 dpv, one group was injected with BTV8/net07 and the other group with BTV-2/SAD2001/01 virulent virus as described for early protection. EDTA-blood and serum were collected to monitor the antibody response and virus load.

### TriDISC vaccination trial in cattle.

Two independent trials were designed to test protection of a cocktail vaccine in cattle. For the first trial, 15 heifers were subcutaneously inoculated with a mixture of the BTV2, -4, and -8 DISC viruses at 3.6 × 10^7^ PFU/animal (3.8 × 10^6^ to 2 × 10^7^ PFU/individual serotype). In addition, 9 animals were mock vaccinated with BTV-free cell lysates. In the second trial, a total of 12 cattle were inoculated with the same mixture of BTV2, -4, and -8 DISC viruses but at a higher dose: 7.5 × 10^7^ PFU/animal (8 × 10^6^ to 3 × 10^7^ PFU/individual serotype). An identical booster injection was given 21 days after the first vaccination. Three weeks after the second vaccination (42 days after the first), the vaccinated animals were segregated into three groups and challenged subcutaneously with 10^5^ TCID_50_s of a virulent isolate of either BTV2 (BTV-2/SAD2001/01 E1/BHK2/KC1), BTV4 (BTV-4/MOR2009/07 KC1), or BTV8 (BTV-8/DE08/BH97/Vero2/BKK1). In parallel, 6 animals were mock vaccinated with BTV-free cell lysates. Whole-blood and serum samples of all animals were taken at regular intervals before and after vaccination and challenge. Clinical data were recorded as described previously (36).

### RNA extraction and RT-qPCR.

RNA extraction from the EDTA-blood samples was performed according to Vandenbussche et al. (37). The triplex RT-qPCR assay included primers and probes for a pan-BTV/S5-specific reaction and for an internal control (IC) and external control (EC) as described by Vandenbussche et al. (37) and was performed on a LightCycler-480 (Roche Diagnostics). For this assay, cycle threshold values (*C_T_* values) of <40.0 were classified as positive, *C_T_* values of ≥40.0 and <45.0 were classified as doubtful, and *C_T_* values of ≥45.0 were considered negative. External standard curves were used for absolute quantification.

Alternatively, viral RNA was extracted from collected blood samples using a commercial extraction kit (Macherey-Nagel), and BTV RNA quantification was achieved by qRT-PCR using the Adiavet BTV detection kit (Adiagene) according to the protocols developed by Toussaint et al. (38). The viral RNA copy numbers of the samples were inferred from a standard curve obtained after specific amplification of a synthetic BTV RNA (segment S1) run in parallel.

### Serology of MultiDISC and TriDISC trials.

Serum samples were analyzed with a commercially available BTV VP7 antibody test kit (competitive ELISA [cELISA]; IDEXX) according to the manufacturer's instructions.

The results were expressed as percentage of negativity compared to the negative kit control (P/N) and transferred to positive (≤70%), doubtful (>70% to <80%) or negative results (≥80%). Serological status was defined as positive (≥30%), doubtful (>25% to <30%), or negative (≤25%).

For detection of neutralizing antibody response in vaccinated animals, a standard seroneutralization (SN) assay was used as described previously (2). Briefly, serum samples were serially diluted 1:2 and added to confluent monolayers of BSR cells in 96-well plates. About 100 infectious BTV particles were added to each well and incubated for 3 days. All dilutions were performed in triplicate for each experiment. The neutralizing titer was defined as the highest dilution of sera allowing complete neutralization of the virus.
